# The millipede genus *Orthomorpha* Bollman, 1893 in Laos (Diplopoda, Polydesmida, Paradoxosomatidae), with descriptions of new species

**DOI:** 10.3897/zookeys.374.6711

**Published:** 2014-01-28

**Authors:** Natdanai Likhitrakarn, Sergei I. Golovatch, Somsak Panha

**Affiliations:** 1 Animal Systematics Research Unit, Department of Biology, Faculty of Science, Chulalongkorn University, Bangkok, 10330, Thailand; 2 Division of Plant Protection, Faculty of Agricultural Production, Maejo University, Chiang Mai, 50290, Thailand; 3 Institute for Problems of Ecology and Evolution, Russian Academy of Sciences, Leninsky pr. 33, Moscow 119071, Russia

**Keywords:** Millipede, *Orthomorpha*, taxonomy, new species, Laos

## Abstract

The genus *Orthomorpha* is currently represented in Laos by nine species, including three, *O. paviei* Brölemann, 1896, *O. communis* Likhitrakarn, Golovatch & Panha, 2011 and *O. cambodjana* (Attems, 1953), which are new to the fauna of the country, and further three new to science: *O. suberectoides* **sp. n.**, *O. gladiata* **sp. n.** and *O. sutchariti* **sp. n.**

## Introduction

The large Southeast Asian millipede genus *Orthomorpha* Bollman, 1893 has recently become the subject of a thorough review (Likhitrakarn et al. 2011), with the result that currently it comprises 51 species ranging from northern Myanmar and Thailand southeastwards to Lombok Island, Indonesia. Among them, only three have hitherto been recorded in Laos: the pantropical anthropochore *Orthomorpha coarctata* (De Saussure, 1860), as well as *Orthomorpha rotundicollis* (Attems, 1937) and *Orthomorpha scabra* Jeekel, 1964, both latter species also shared with Vietnam (Likhitrakarn et al. 2011, 2014).

The present paper puts on record a few more *Orthomorpha* from Laos, including three species heretofore known from the adjacent parts of Indochina (one also from as far south as Indonesia), and further three new to science.

## Material and methods

The material was collected during a field trip to southern Laos undertaken in 2013 by SP and members of the Animal Systematics Research Unit, Chulalongkorn University. Live animals were photographed on the spot. Specimens were preserved in 75% ethanol, and morphological investigations were carried out in the laboratory using an Olympus stereomicroscope. Scanning electron micrographs (SEM) of gonopods coated with gold were taken using a JEOL, JSM–5410 LV microscope, returned to alcohol upon examination. Digital images of the specimens were taken in the laboratory and assembled using the “Cell^D^” automontage software of the Olympus Soft Imaging Solution GmbH package. In addition, line drawings of gonopods were also prepared. All material is housed in the Museum of Zoology, Chulalongkorn University (CUMZ), Bangkok, Thailand.

Collecting sites were located by GPS using the WGS84 datum.

In the catalogue sections, D stands for the original description, subsequent descriptive notes or appearance in a key while M for a mere mention.

## Taxonomic part

### Order Polydesmida
Family Paradoxosomatidae Daday, 1889
Genus *Orthomorpha* Bollman, 1893

#### Orthomorpha paviei

Brölemann, 1896

http://species-id.net/wiki/Orthomorpha_paviei

Figs 1 – 3

Orthomorpha Paviei Brölemann, 1896: 1 (D).
Orthomorpha Paviei – Brölemann 1904: 8 (D).
Prionopeltis Paviei ? – Attems 1914: 204 (M).
Pratinus paviei – Attems 1937: 122 (M).
Orthomorpha paviei – Jeekel 1963: 265 (M); 1964: 359 (M); 1968: 56 (M); Golovatch 1998: 42 (D); Enghoff et al. 2004: 34 (M); Enghoff 2005: 97 (M); Likhitrakarn et al. 2011: 52 (D).

##### Material examined:

1 ♂ (CUMZ), Laos, Champasak Province, Khong District, Khone Phapheng Waterfall, 82 m a.s.l., 13°57'47"N, 105°59'17"E, 23.07.2013, leg. W. Siriwut.

##### Diagnosis.

According to the key in Likhitrakarn et al. (2011), this species is especially similar to *Orthomorpha coarctata* (De Saussure, 1860), but differs in the paraterga more strongly developed, in having two, fully separated, sternal cones between ♂ coxae 4, and a nearly trifid gonopod tip with two clearly larger prongs being deeply split with a third, very small denticle lying ventrally at about midway of the second, slightly stronger prong.

##### Redescription.

Length 25 mm (♂), width of midbody pro- and metazona 2.06 and 3.23 mm, respectively.

**Figure 1.**
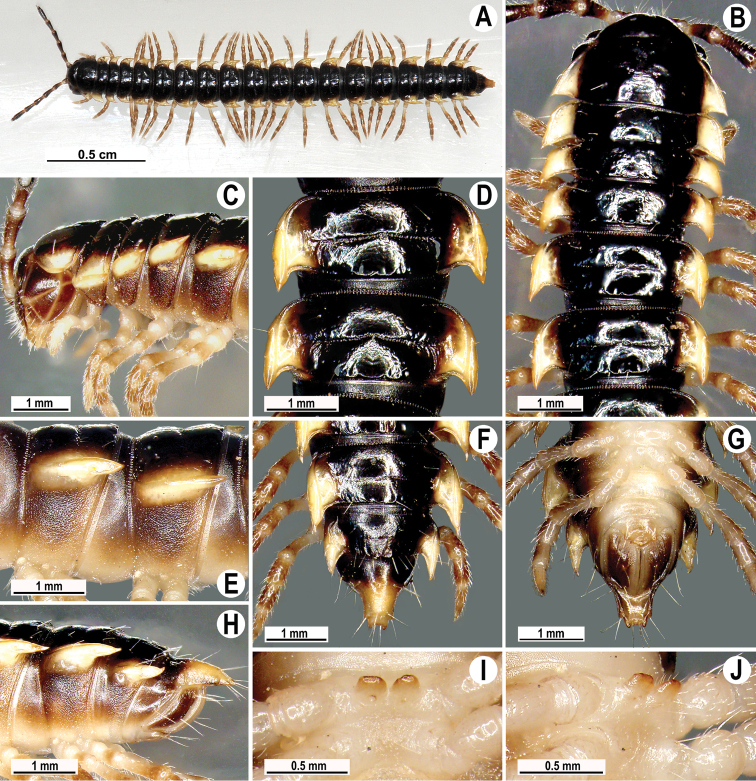
*Orthomorpha paviei* Brölemann, 1896, ♂ from Laos. **A** habitus, live coloration; **B**, **C** anterior part of body, dorsal and lateral views, respectively**D**, **E** segments 10 and 11, dorsal and lateral views, respectively **F–H** posterior part of body, dorsal, ventral and lateral views, respectively**I**, **J** sternal cones between coxae 4, subcaudal and sublateral views, respectively.

Coloration of live animals blackish (Fig. 1A), paraterga and epiproct contrasting dark yellow, head and antennae brownish, legs pale brownish; coloration in alcohol, after three months of preservation, faded to black-brown (Fig. 1B–H), paraterga and epiproct pale whitish yellow or pale brown, legs whitish to pale brown distally.

Clypeolabral region densely setose, vertigial region with a few setae only; epicranial suture distinct. Antennae long (Fig. 1A), reaching posterior end of body segment 4 when stretched dorsally. In width, head < segment 3 = 4 < collum < segment 2 < 5–17, gently and gradually tapering thereafter. Collum with three transverse rows of setae: 4+4 in anterior, 3+3 in intermediate, and 1+1 in posterior row; a very faint incision laterally near midway; caudal corner of paraterga pointed, dentiform, slightly upturned, but not drawn behind rear margin (Fig. 1B, C).

Tegument smooth and shining, prozona finely shagreened, metaterga smooth and delicately rugulose, leathery; surface below paraterga finely microgranulate. Postcollum metaterga with two transverse rows of setae traceable at least as insertion points when setae broken off: 2+2 in anterior (pre-sulcus), 3–4+3–4 in posterior (post-sulcus) row. Tergal setae long, strong, slender, about 1/3 of metatergal length. Axial line visible only on metaterga, slightly better developed on their posterior halves. Paraterga very strongly developed (Fig. 1B–H), mostly slightly upturned, all lying faintly below dorsum, set at about upper 1/3 of midbody height, subhorizontal, caudal corner almost or fully pointed, increasingly well spiniform and produced behind rear tergal margin until segment 18; paraterga very thin in lateral view, blunt blades, modestly enlarged in pore-bearing segments, thinner in poreless ones. Calluses on paraterga 2–4 delimited by a sulcus only dorsally, on following paraterga both dorsally and ventrally. Paraterga 2 broad, anterior edge angular, lateral edge with one larger and two smaller, but evident incisions in anterior 1/3; posterior edge well concave (Fig. 1B, D, F). Anterior edges of paraterga 3–9 clearly convex, of paraterga 10–18 nearly straight and slightly bordered. Lateral edge of paraterga with two slight, but evident incisions, one in anterior 1/3, the other in posterior 1/3. Posterior edge of paraterga clearly concave, especially well so in segments 16–19 (Fig. 1F–H). Ozopores evident, lateral, lying in an ovoid groove at about 1/4 of metatergite’s length in front of caudal corner. Transverse sulcus usually distinct (Fig. 1C, E, H), complete on metaterga 5–19, incomplete and nearly wanting on 19^th^, shallow, nearly reaching bases of paraterga, rather faintly beaded at bottom. Stricture between pro- and metazona wide, clearly beaded at bottom down to base of paraterga (Fig. 1B, D, F). Pleurosternal carinae complete crests with a sharp caudal tooth in segment 2, a very sharp, caudal tooth in segments 3 and 4, a small, mostly sharp tooth in segments 5–9 (Fig. 1C, E). Epiproct (Fig. 1F, G) conical, flattened dorsoventrally, with two evident apical papillae; tip subtruncate; pre-apical papillae small, but visible, lying rather close to tip. Hypoproct roundly subtriangular, setiferous knobs at caudal edge evident and well-separated.

Sterna sparsely setose, without modifications; two rather large, fully separated, sternal cones between ♂ coxae 4 (Fig. 1I, J). A paramedian pair of evident tubercles in front of gonopod aperture. Legs moderately long and slender, midbody ones ca 1.1–1.2 times as long as body height, prefemora without modifications, ♂ tarsal brushes present until legs of segment 8.

**Figure 2.**
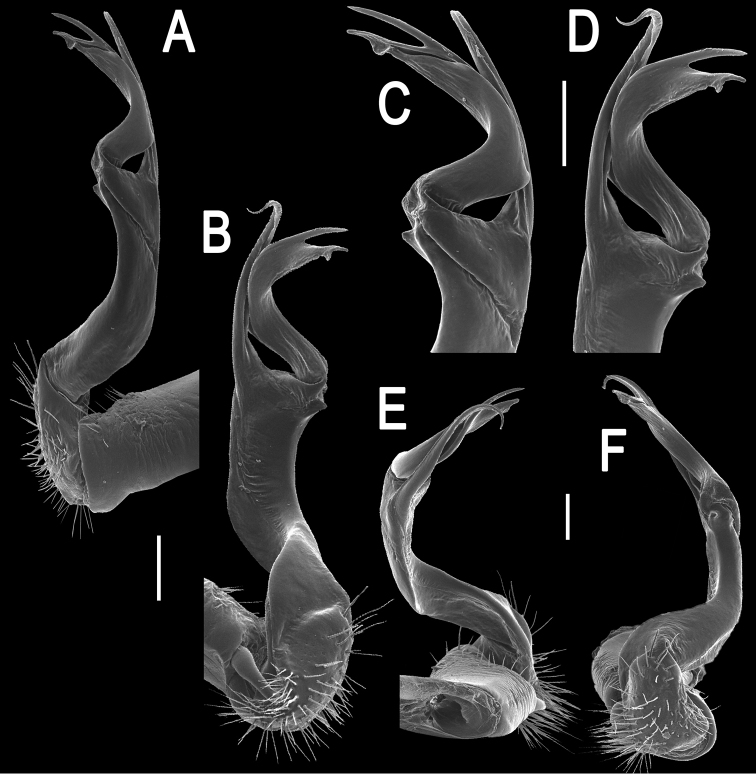
*Orthomorpha paviei* Brölemann, 1896, ♂ from Laos, left gonopod. **A**, **B** lateral and mesal views, respectively **C**, **D** telopodite, lateral and mesal views, respectively **E**, **F** distal part, subcaudal and suboral views, respectively. Scale bar: 0.2 mm.

**Figure 3.**
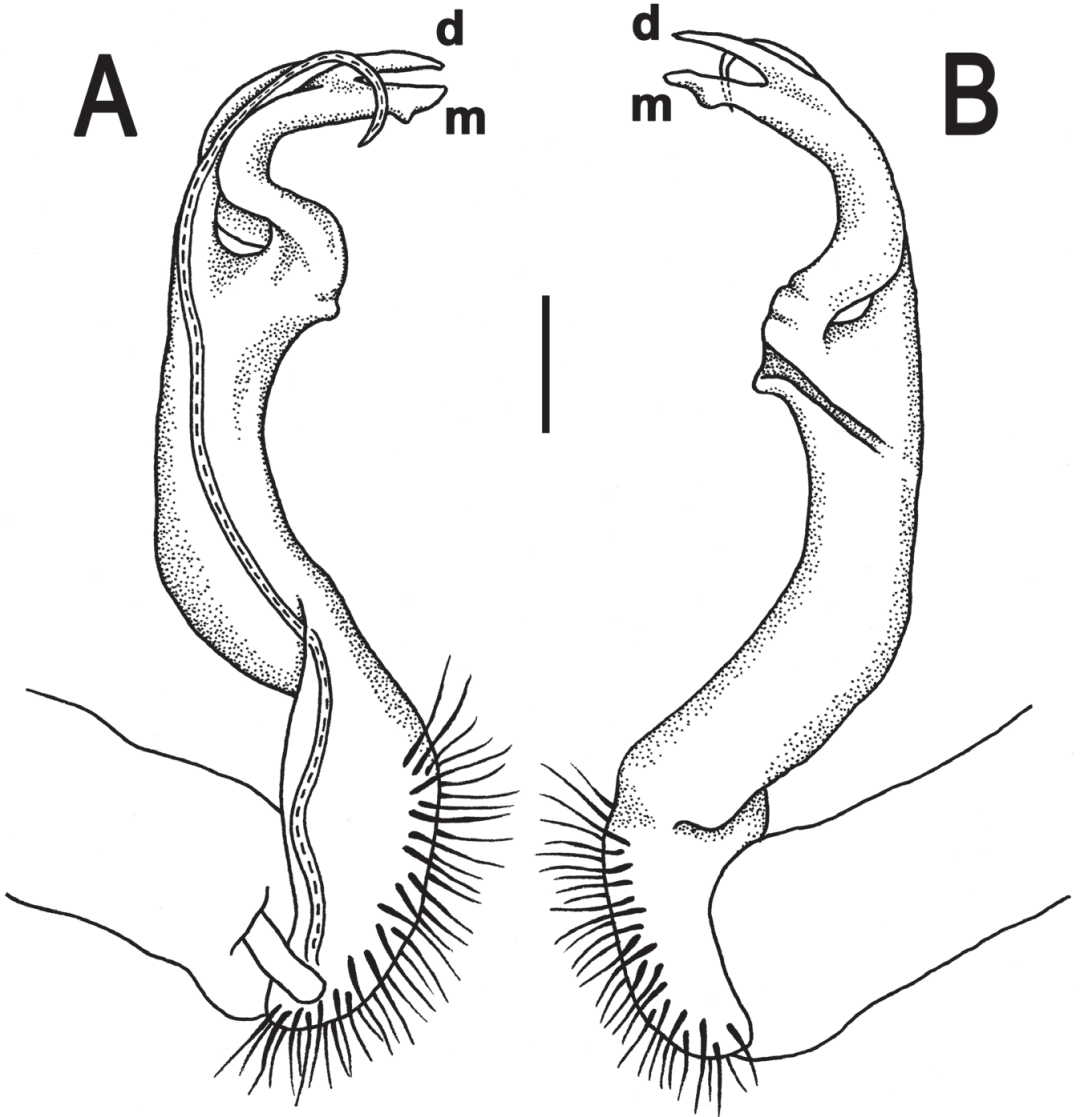
*Orthomorpha paviei* Brölemann, 1896, ♂. **A**, **B** left gonopod, mesal and lateral views, respectively. Scale bar: 0.5 mm.

Gonopods (Figs 2, 3) with slender and long coxae, the latter with several setae distoventrally. Femorite about 3 times as long as prefemoral (= strongly setose) part. Femorite slender, slightly curved, postfemoral portion demarcated by an oblique lateral sulcus; tip of solenophore indistinctly trifid, both dorsal (**d**) and middle (**m**) prongs being sharp and deeply split, middle prong slightly stronger, supplied with a minute, but evident subterminal knob/lobule ventrally at about midway; solenomere long and flagelliform.

##### Remarks.

The new specimen agrees nearly fully with the most detailed and beautifully illustrated descriptions of the species given by Brölemann (1896, 1904), but the holotype is larger: body length 40 mm versus 25 mm, width of midbody metazona 4.25 mm versus 3.23 mm. In addition, a transverse sulcus is visible in segments 4–18 in the holotype versus segments 5–19 in the fresh ♂. Originally described from an unspecified locality in Thailand (Brölemann 1896, 1904), *Orthomorpha paviei* is thus new to the fauna of Laos.

In having both apical prongs of the gonopods rather deeply split, this species is somewhat intermediate between the genera *Antheromorpha* Jeekel, 1968 and *Orthomorpha* Bollman, 1893 (Likhitrakarn et al. 2011).

The above detailed redescription is given not only to better outline the quite modest variations found in *Orthomorpha paviei*, but also to serve as a pattern for further, more condensed descriptions or descriptive notes below.

#### Orthomorpha cambodjana

(Attems, 1953)

http://species-id.net/wiki/Orthomorpha_cambodjana

Figs 4 – 6

Pratinus cambodjanus Attems 1953: 168 (D).
Orthomorpha cambodjana – Jeekel 1963: 265 (M); 1964: 361 (M, D); 1968: 56 (M); Hoffman 1977: 700 (M); Golovatch 1998: 42 (M, D); Likhitrakarn et al. 2011: 66 (D).

##### Material examined.

2 ♂ (CUMZ), Laos, Attapu Province, Xaysetha District, Ban Kasom, 116 m a.s.l., 15°06'27"N, 106°51'14"E, 16.10.2013, leg. C. Sutcharit and W. Siriwut.

##### Descriptive notes.

Length 32–35 mm (♂), width of midbody pro- and metazona 2.6–2.7 and 3.8–3.9 mm (♂), respectively.

**Figure 4.**
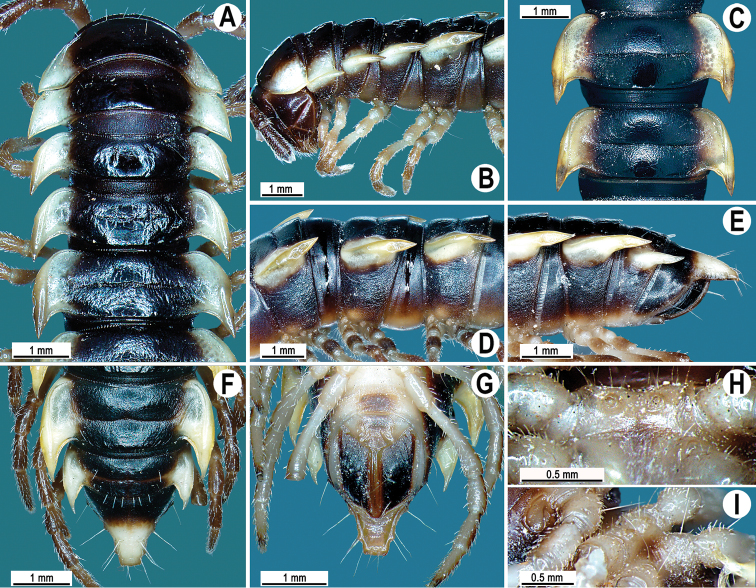
*Orthomorpha cambodjana* (Attems, 1953), ♂. **A**, **B** anterior part of body, dorsal and lateral views, respectively; **C** segments 10–11, dorsal view **D** segments 9–11, lateral view **E–G** posterior part of body, lateral, dorsal and ventral views, respectively; **H, I** sternal cones between coxae 4, subcaudal and sublateral views, respectively.

Coloration in alcohol, after one month of preservation, blackish (Fig. 4A–F), paraterga and epiproct light yellow to whitish yellow; venter, legs and antennae whitish to pale brown distally (Fig. 4A–I).

Antennae reaching segment 3 when stretched dorsally. Paraterga very strongly developed (Fig. 4A–G), anterolateral edge clearly convex. Calluses on paraterga 2 and 3 delimited by a sulcus only dorsally, following paraterga by a sulcus both dorsally and ventrally, strongly bordered. Pleurosternal carinae complete crests only in segment 2 (Fig. 4B, D, E), a small, sharp, caudal tooth in segments 3–7, a very faint tubercle until segment 15. Legs long and slender, midbody ones ca 1.1–1.3 times as long as body height, prefemora without modifications, tarsal brushes present until ♂ leg 7.

**Figure 5.**
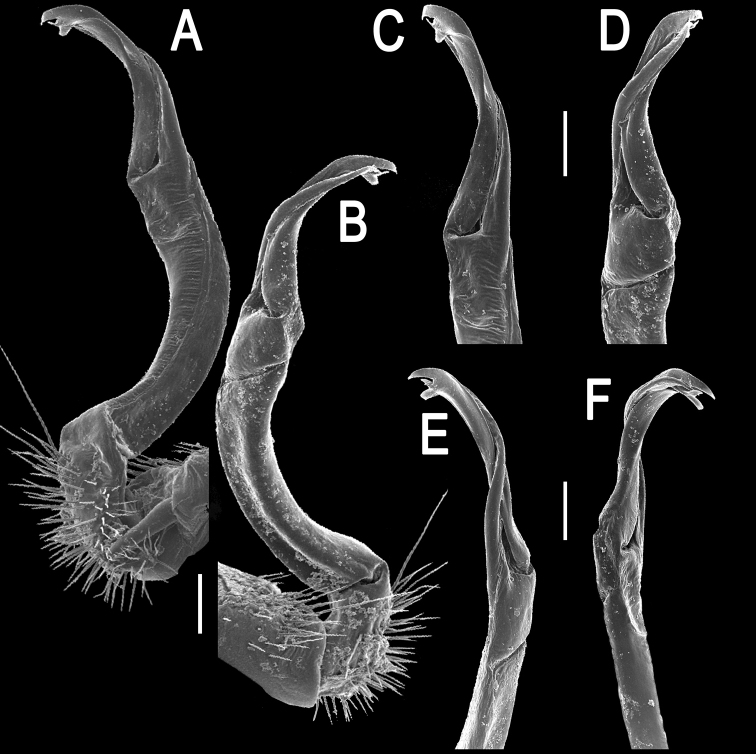
*Orthomorpha cambodjana* (Attems, 1953), ♂, right gonopod. **A**, **B** mesal and lateral views, respectively **C–F** distal part of left gonopod, sublateral, submesal, subcaudal and suboral views, respectively. Scale bar: 0.2 mm.

**Figure 6.**
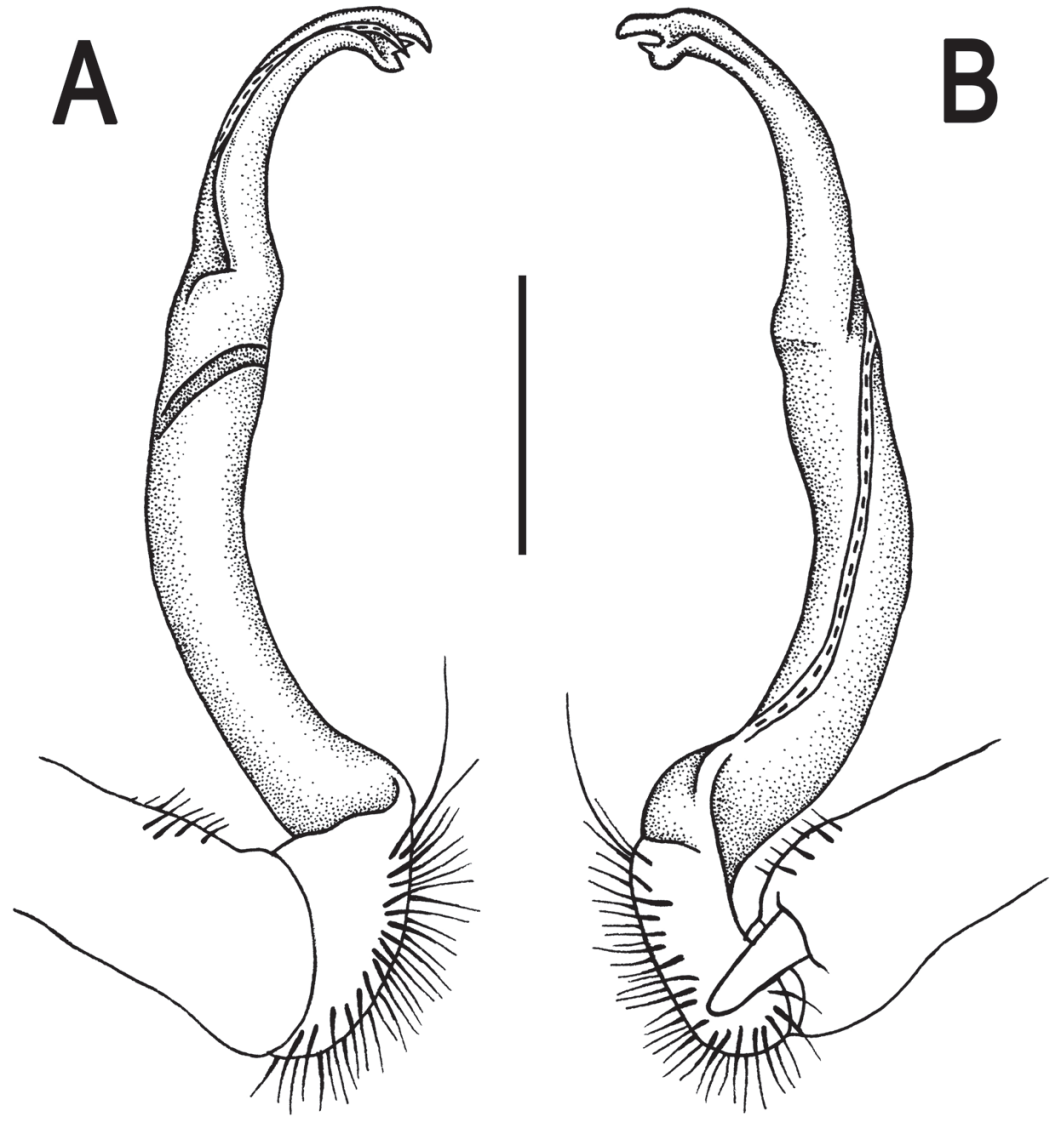
*Orthomorpha cambodjana* (Attems, 1953), ♂. **A**, **B** right gonopod, lateral and mesal views, respectively. Scale bar: 0.5 mm.

Gonopod solenophore with a tridentate tip (Figs 5, 6), dorsalmost prong being subacuminate, longer than both others, whereas middle denticle shortest and pointed.

##### Remarks.

This species has hitherto been known only from a ♂ lectotype and a ♀ paralectotype, currently both rather strongly faded, taken at Sre-Umbell, Kampot Province, southern Cambodia (Attems 1953, Likhitrakarn et al. 2011). The above fresh samples show only slight variations, mainly a darker coloration, as opposed to the types which have recently been redescribed in due detail (Likhitrakarn et al. 2011). This species is thus new to the fauna of Laos, suggesting a far wider distribution in Indochina.

#### Orthomorpha communis

Likhitrakarn, Golovatch & Panha, 2011

http://species-id.net/wiki/Orthomorpha_communis

Orthomorpha communis Likhitrakarn, Golovatch & Panha 2011: 37 (D).

##### Material examined.

1 ♂, 2 ♀ (CUMZ), Laos, Savannakhet Province, Atsaphangthong District, Ban Than Bang, 170 m, 16°42'12"N, 105°12'06"E, 19.10.2013, leg. C. Sutcharit and W. Siriwut.

##### Descriptive notes.

Length 34 mm (♂) or 36–37 mm (♀), width of midbody pro- and metazona 2.9 and 4.5 mm (♂), 3.0–3.8 and 4.5–5.3 mm (♀), respectively.

##### Remarks.

The above material fully agrees with the original, quite detailed description of this species (Likhitrakarn et al. 2011). It appears to be rather widespread not only in the eastern part of Thailand close to the frontier to Cambodia, but also in southern Laos. This species is new to the fauna of Laos, again suggesting a much wider distribution in Indochina.

#### Orthomorpha suberectoides sp. n.

http://zoobank.org/695749B1-EFAF-4D08-BF0E-2C817DC63C95

http://species-id.net/wiki/Orthomorpha_suberectoides

Figs 7 – 9

##### Holotype

♂ (CUMZ), Laos, Attapu Province, Xaysetha District, Ban Lak No. 52, 224 m a.s.l., 15°09'24"N, 106°44'01"E, 16.10.2013, leg. C. Sutcharit.

##### Name.

To emphasize the apparent similarity to *Orthomorpha suberecta* Likhitrakarn, Golovatch & Panha, 2011; adjective.

##### Diagnosis.

This new species strongly resembles *Orthomorpha suberecta*, especially as regards the shape of the paraterga, but differs by a larger body, in the paraterga being broader, the metatergal tegument clearly rugose, and the pleurosternal carinae more strongly developed.

##### Description.

Length 35 mm (♂), width of midbody pro- and metazona 3.5 and 5.0 mm, respectively.

**Figure 7.**
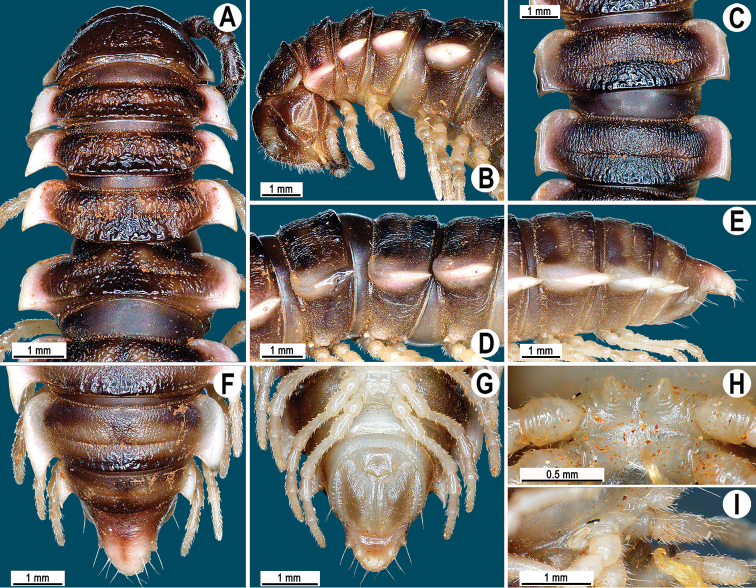
*Orthomorpha suberectoides* sp. n., ♂ holotype. **A**, **B** anterior part of body, dorsal and lateral views, respectively **C** segments 10–11, dorsal view **D** segments 9–11, lateral view **E–G** posterior part of body, lateral, dorsal and ventral views, respectively **H, I** sternal cones between coxae 4, subcaudal and sublateral views, respectively.

Coloration in alcohol, after one month of preservation, dark brown (Fig. 7A–F), paraterga and epiproct pale pinkish or pallid, head and antennae brownish, venter and legs pale whitish to pale brown (Fig. 7B–I).

All other characters as in *Orthomorpha paviei*, except as follows.

Antennae (Fig. 7A) reaching posterior end of body segment 3 when stretched dorsally. In width, head < collum < segment 2 < 3 < 4 < 5–16, gently and gradually tapering thereafter. Collum with three transverse rows of setae: 4+4 in anterior, 2+2 in intermediate, and 4+4 in caudal row; paraterga slightly declivous, broadly rounded and narrowly bordered, caudal corner pointed, dentiform, but not drawn behind rear margin (Fig. 7A, B).

Tegument shining and rugulose (Fig. 7A–F), metaterga rather obviously rugose, leathery, with traces of tubercles/wrinkles; surface below paraterga microgranulate and rugulose. Postcollum metaterga with two transverse rows of setae: 2+2, mostly abraded setae in anterior (pre-sulcus) row, 3+3 in posterior (post-sulcus) one, these setae or their traces being borne on evident cones growing stronger laterally, until segment 6 visible only as insertion points, tuberculations gradually growing smaller thereafter. Tergal setae simple, rather long, about 1/3 of metatergal length, mostly abraded. Axial line traceable, especially clear on collum and following metaterga. Paraterga very strongly developed (Fig. 7A–G), mostly slightly upturned, all lying faintly below dorsum, set at about half of midbody height, subhorizontal, in lateral view modestly enlarged in pore-bearing segments, thinner in poreless ones; shoulders broadly rounded, narrowly bordered, fused to callus; caudal corner almost or fully pointed, lying within rear tergal margin, after segment 16 drawn increasingly well beyond it, slightly curved mesad on segments 17–19 (Fig. 7E–G). Calluses delimited by a sulcus only dorsally. Paraterga 2 broad, anterior edge convex, lateral edge with three small and acute denticles, the one near caudal corner being particularly small (Fig. 7A, B). Each following poreless segment with two incisions, each pore-bearing one with one, often evident incision in front of ozopore. Posterior edge of paraterga slightly concave, especially clearly so in segments 16–19. Ozopores evident, lateral, lying in an ovoid groove at about 1/4 of metatergite’s length in front of caudal corner. Transverse sulcus usually distinct (Fig. 7A–F), complete on metaterga 5–19, incomplete on segment 4, narrow, not reaching bases of paraterga, ribbed at bottom. Stricture between pro- and metazona narrow, clearly ribbed at bottom down to base of paraterga (Fig. 7A–F). Pleurosternal carinae complete crests with a sharp caudal tooth in segments 2–7, a very sharp, caudal tooth in segments 8–17, a small, rather sharp tooth in segment 18 (Fig. 7B, D, E). Epiproct (Fig. 7F, G) conical, flattened dorsoventrally, with two evident apical papillae, both latter directed ventrocaudally and acute at tip; pre-apical papillae small denticles lying close to tip. Hypoproct (Fig. 7G) roundly subtriangular, setiferous knobs at caudal edge evident and well-separated.

Sterna sparsely setose, without modifications; cross-impressions shallow; a paramedian pair of evident, fully separated, small, sternal cones between ♂ coxae 4 (Fig. 7H, I). A paramedian pair of evident tubercles in front of gonopod aperture. Legs moderately long and slender, midbody ones ca 1.0–1.1 times as long as body height, prefemora without modifications, ♂ tarsal brushes absent.

**Figure 8.**
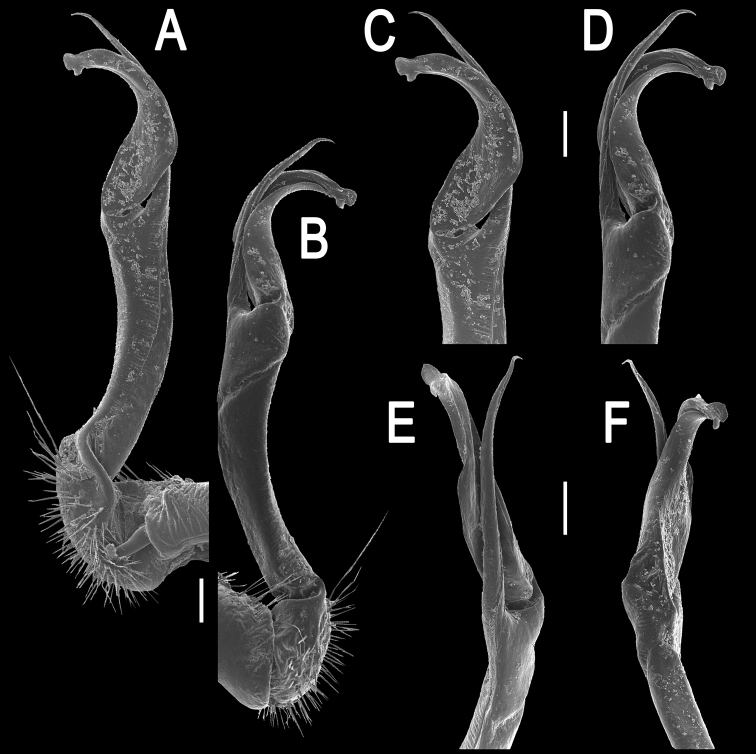
*Orthomorpha suberectoides* sp. n., ♂ holotype, right gonopod. **A**, **B** mesal and lateral views, respectively **C–F** distal part of left gonopod, mesal, lateral, suboral and subcaudal views, respectively. Scale bar: 0.2 mm.

**Figure 9.**
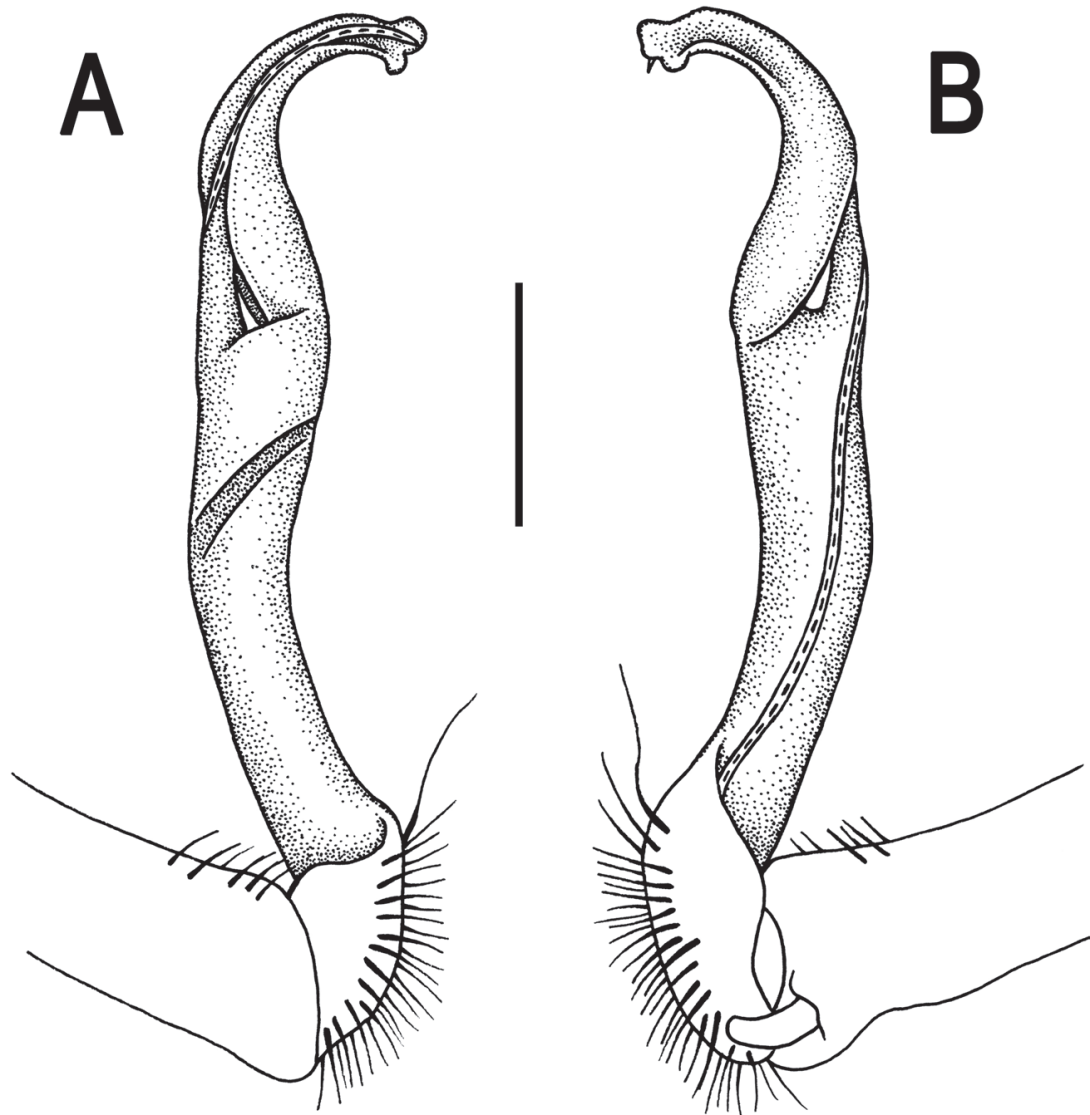
*Orthomorpha suberectoides* sp. n., ♂ holotype. **A**, **B** right gonopod, lateral and mesal views, respectively. Scale bar: 0.5 mm.

Gonopods (Figs 8, 9) with slender and long coxae, the latter with several setae distoventrally. Femorite about 3 times as long as prefemoral portion. Femorite slender, slightly curved, postfemoral portion demarcated by an oblique lateral sulcus. Solenophore with *lamina lateralis* clearly smaller than *lamina medialis*, tip distinctly bilobed, dorsal lobule larger; solenomere long and flagelliform.

##### Remarks.

According to the key in Likhitrakarn et al. (2011), this new species seems to be especially similar to *Orthomorpha suberecta*. The latter taxon has originally been found near Cave Suwannnakhuha, Suwannnakhuha District, Nong Bua Lamphu Province, northeastern Thailand (Likhitrakarn et al. 2011), thus very far away (550 km) from the type locality of *Orthomorpha suberectoides* sp. n. Both these species are thus distinct not only morphologically (see Diagnosis), but also geographically.

#### Orthomorpha gladiata sp. n.

http://zoobank.org/B785592D-54B8-4C4D-918A-F43D9ED88C4A

http://species-id.net/wiki/Orthomorpha_gladiata

Figs 10 – 12

##### Holotype

♂ (CUMZ), Laos, Champasak Province, Paksong District, Tadedu, 906 m a.s.l., 15°11'35"N, 106°06'07"E, 16.10.2013, leg. S. Panha and C. Sutcharit.

##### Paratype.

1 ♂ (CUMZ), same data, together with holotype.

##### Name.

To emphasize the sword-shaped tip of the gonopod; adjective.

##### Diagnosis.

*Orthomorpha gladiata* sp. n. seems to be especially similar to *Orthomorpha hydrobiologica* Attems, 1930, a species known to range from eastern Java, Indonesia to Indochina (Likhitrakarn et al. 2011), but differs clearly in the tip of the solenophore being nearly monodentate and sword-shaped.

##### Description.

Length 25–27 mm (♂), width of midbody pro- and metazona 1.7–1.9 and 2.8–2.9 mm, respectively.

**Figure 10.**
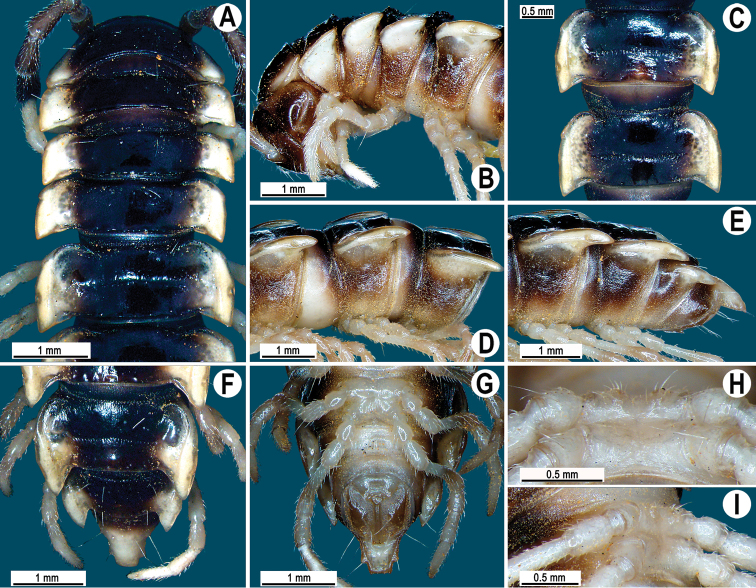
*Orthomorpha gladiata* sp. n., **A–B** ♂ paratype **C–I** ♂ holotype. **A**, **B** anterior part of body, dorsal and lateral views, respectively **C** segments 10–11, dorsal view **D** segments 9–11, lateral view **E–G** posterior part of body, lateral, dorsal and ventral views, respectively **H**, **I** sternal cones between coxae 4, subcaudal and sublateral views, respectively.

Coloration in alcohol, after one month of preservation, dark brown (Fig. 10A–G), paraterga and epiproct light yellow or pale yellowish, head, surface below paraterga and antennae brownish, venter and legs whitish to pale brown (Fig. 10A–I).

All other characters as in *Orthomorpha paviei*, except as follows.

Antennae (Fig. 10A) reaching body segment 3 when stretched dorsally. In width, head < collum < segments 3 and 4 < 2 = 5–16, gently and gradually tapering thereafter. Collum with three transverse rows of setae: 3+3 in anterior, 2+2 in intermediate, and 3+3 in posterior row; caudal corner of paraterga very narrowly rounded, slightly upturned, but not drawn behind rear margin (Fig. 10A, B).

Postcollum metaterga with two transverse rows of setae traceable at least as insertion points when setae broken off: 2+2 in anterior (pre-sulcus) and 3+3 in posterior (post-sulcus) row, these setae being borne on small cones growing stronger laterally. Metaterga 17–19 with 4+4 setae in posterior row, likewise borne on evident cones growing stronger laterally. Axial line rather difficult to see, but traceable both on pro- and metaterga. Paraterga very strongly developed (Fig. 10A–G), mostly slightly upturned, all lying faintly below dorsum, set at about upper 1/4 of midbody height, mostly subhorizontal, caudal corner narrowly rounded to nearly pointed; paraterga very thin in lateral view, modestly enlarged in pore-bearing segments, thinner in poreless ones. Calluses on paraterga 2–4 delimited by a sulcus only dorsally, on following paraterga both dorsally and ventrally. Paraterga 2 broad, anterior edge rounded, lateral edge with one larger and one smaller, but evident incision in anterior 1/3; posterior edge clearly concave (Fig. 10A, C, F). Anterior edges of paraterga broadly rounded and narrowly bordered, fused to callus. Lateral edge of paraterga with two slight, but evident incisions, one in anterior 1/3, the other in posterior 1/3. Posterior edge of paraterga clearly concave, lateral corner extending increasingly beyond rear tergal margin, especially strongly so in segments 17–19 (Fig. 10F, G). Transverse sulcus usually distinct (Fig. 10A, C, F), complete on metaterga 5–18, incomplete in segments 4 and 19, rather deep, reaching bases of paraterga, rather clearly ribbed at bottom. Stricture between pro- and metazona narrow, clearly ribbed at bottom down to base of paraterga (Fig. 10A–F). Pleurosternal carinae complete crests with a sharp caudal tooth in segments 2–4, a very sharp, caudal tooth in segments 5–7, a small, mostly sharp tooth until segment 16 (Fig. 10B, D, E). Epiproct (Fig. 10F, G) conical, flattened dorsoventrally, with two evident apical papillae; tip subtruncate; pre-apical papillae small, but visible, lying rather close to tip.

Two small, low, rounded, fully separated, sternal cones between ♂ coxae 4 (Fig. 10H, I). A paramedian pair of evident tubercles in front of gonopod aperture. Legs rather long and slender, midbody ones ca 1.2–1.4 times as long as body height, prefemora without modifications, ♂ tarsal brushes present only on legs 1–3.

**Figure 11.**
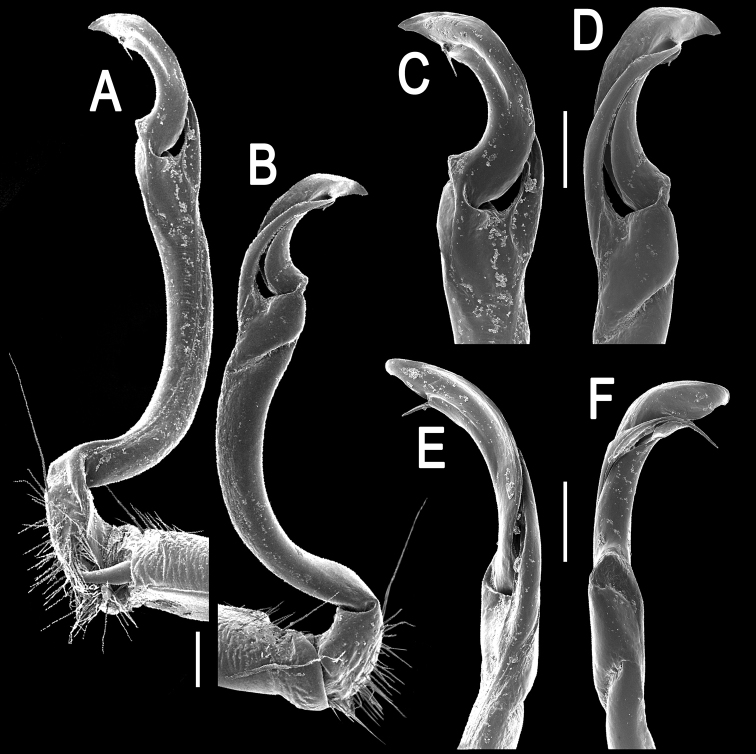
*Orthomorpha gladiata* sp. n., ♂ holotype, right gonopod. **A**, **B** mesal and lateral views, respectively **C–F** distal part of left gonopod, mesal, lateral, suboral and subcaudal views, respectively. Scale bar: 0.2 mm.

**Figure 12.**
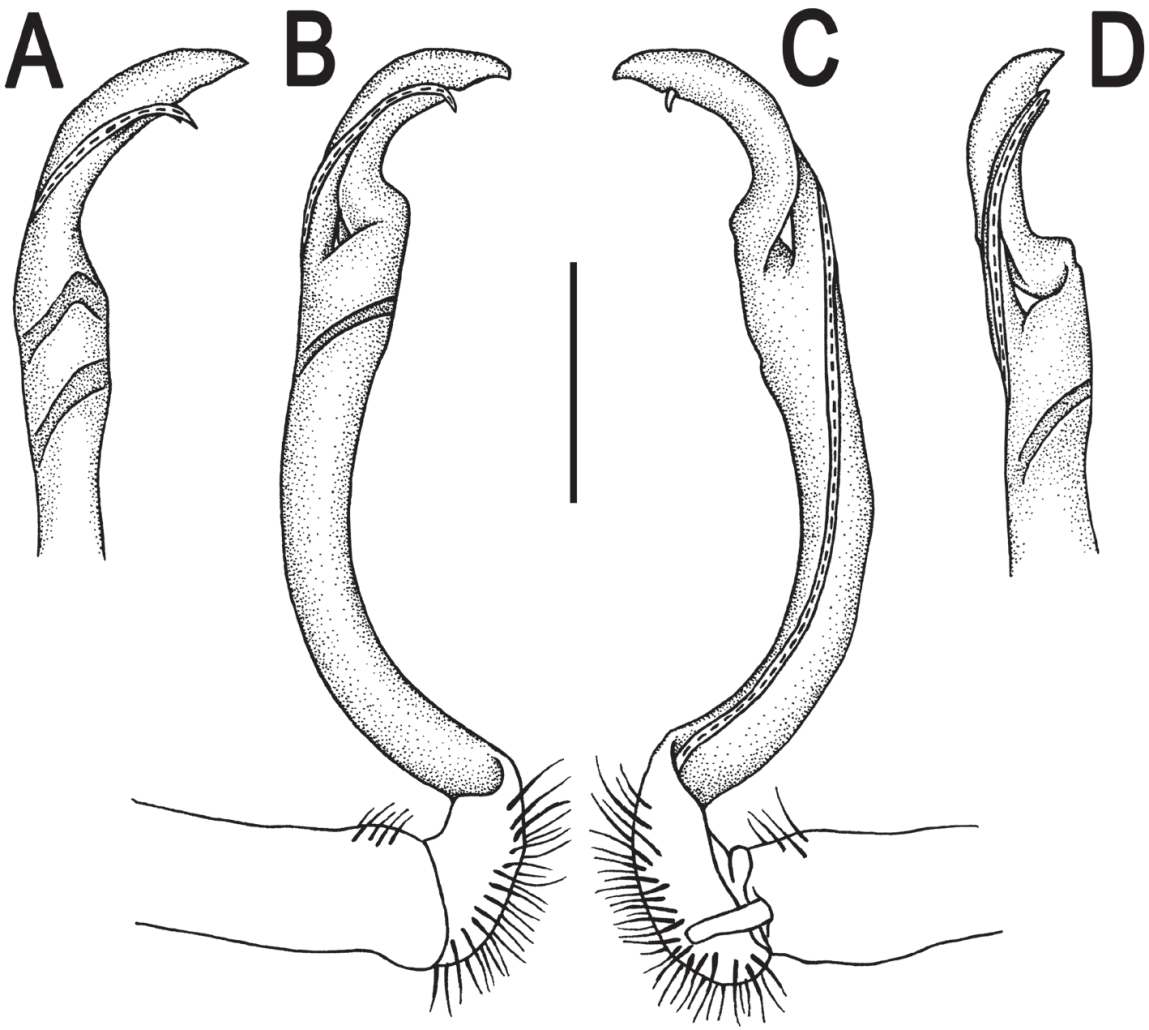
*Orthomorpha gladiata* sp. n., ♂ holotype. **A–D** right gonopod, suboral, lateral, mesal and subcaudal views, respectively. Scale bar: 0.5 mm.

Gonopods (Figs 11, 12) with slender and long coxae, the latter with several setae distoventrally. Femorite about 3 times as long as prefemoral portion. Femorite very slender, slightly curved, postfemoral portion demarcated by an oblique lateral sulcus. Solenophore with a nearly monodentate and sword-shaped tip; solenomere long and flagelliform.

##### Remarks.

The gonopod tip of *Orthomorpha gladiata* sp. n. is much like in *Orthomorpha parasericata* Likhitrakarn, Golovatch & Panha, 2010, from southern Thailand (Likhitrakarn et al. 2010, 2011), but the new species differs clearly not only in some somatic characters (e.g. the considerably broader paraterga), but also by the presence of an evident oblique sulcus demarcating a gonopod postfemoral portion.

#### Orthomorpha sutchariti sp. n.

http://zoobank.org/D5E01463-1933-4A03-97C6-2D678FEAB8D2

http://species-id.net/wiki/Orthomorpha_sutchariti

Figs 13 – 15

##### Holotype

♂ (CUMZ), Laos, Champasak Province, Paksong District, Tadedu, 906 m a.s.l., 15°11'35"N, 106°06'07"E, 16.10.2013, leg. S. Panha and C. Sutcharit.

##### Paratype.

1 ♀ (CUMZ), same data, together with holotype.

##### Name.

To honour Dr. Chirasak Sutcharit, Professor at the Department of Biology of Chulalongorn University, Bangkok, who participated in collecting the type specimens and taught the first author the basics of taxonomy.

##### Diagnosis.

Differs in the colour pattern which has lighter caudal halves of the metaterga, the mostly strongly elevated and laterally bordered paraterga, and a trifid solenophore showing a subacuminate, longest, dorsal prong.

##### Description.

Length ca 42 mm (♂) or 44.5 mm (♀), width of midbody pro- and metazona 3.4 and 4.8 mm (♂), 3.5 and 5.6 mm (♀), respectively.

**Figure 13.**
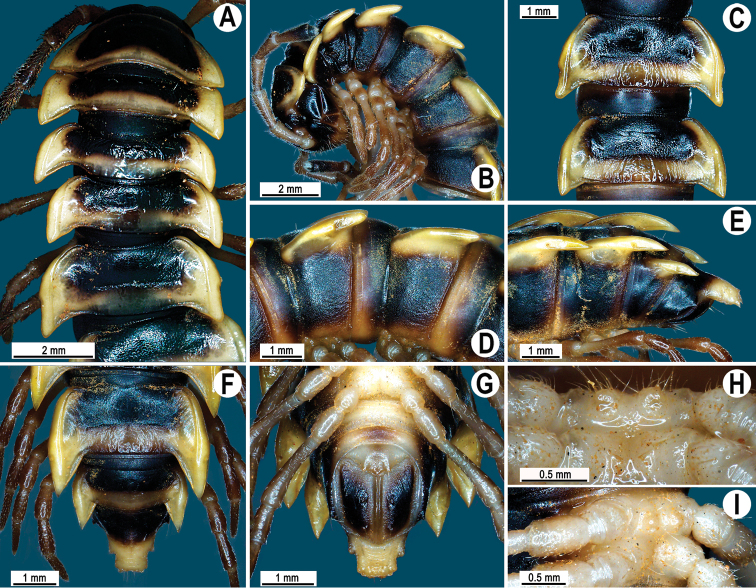
*Orthomorpha sutchariti* sp. n., ♂ holotype. **A**, **B** anterior part of body, dorsal and lateral views, respectively **C**, **D** segments 10–11, dorsal and lateral views, respectively **E–G** posterior part of body, lateral, dorsal and ventral views, respectively **H**, **I** sternal cones between coxae 4, subcaudal and sublateral views, respectively.

Coloration in alcohol, after one month of preservation, rather uniformly dark brown (Fig. 13A–G) with lighter caudal halves of metaterga, paraterga and epiproct contrasting light yellow, mid-dorsal regions of prozona strongly infuscate, blackish like most of metaterga; antennae and legs brown to light brown (Fig. 13A–I).

All other characters as in *Orthomorpha paviei*, except as follows.

Antennae (Fig. 13A, B) surpassing posterior end of body segment 3 (♂) or 2 (♀) when stretched dorsally. In width, head < collum < segment 3 = 4 < 2 < 5–15, gently and gradually tapering thereafter. Collum with three transverse rows of setae: 4+4 in anterior, 2+2 in intermediate, and 4+4 in posterior row; a very faint incision laterally near midway; caudal corner of paraterga very narrowly rounded, slightly upturned, but not drawn behind rear margin (Fig. 13A, B).

Tegument smooth and shining, metaterga smooth and delicately rugulose, leathery, posterior halves of metaterga rugose, with traces of tubercles/wrinkles; surface below paraterga finely microgranulate. Postcollum metaterga with two transverse rows of setae traceable at least as insertion points when setae broken off: 2+2 in anterior (pre-sulcus), 3+3 in posterior (post-sulcus) row. Axial line faint, but traceable both on pro- and metaterga. Paraterga very strongly developed (Fig. 13A–G), mostly slightly upturned, lying either faintly above (♂) or slightly below dorsum (♀), set at about upper 1/3 of midbody height, subhorizontal (♀), caudal corner very narrowly rounded, extended beyond rear tergal margin, increasingly well produced caudally in segments 16–19 (Fig. 13F, G); paraterga very thin in lateral view, blunt blades, modestly enlarged in pore-bearing segments, thinner in poreless ones. Calluses on paraterga 2 and 3 delimited by a sulcus only dorsally, on following paraterga both dorsally and ventrally. Paraterga 2 broad, anterior edge angular, lateral edge with one stronger and two smaller, but evident incisions in anterior 1/3; posterior edge clearly oblique (Fig. 13A, C, F). Anterior edges of paraterga broadly rounded and narrowly bordered, fused to callus. Transverse sulcus usually distinct (Fig. 13A, C, F), complete on metaterga 5–18, incomplete and nearly wanting on segments 4 and 19, shallow, not reaching bases of paraterga, rather faintly ribbed at bottom. Stricture between pro- and metazona narrow, clearly ribbed at bottom down to base of paraterga (Fig. 13A–F). Pleurosternal carinae complete crests with a sharp caudal tooth in segments 2–7 (♂) or 2–4 (♀), a very sharp, caudal tooth in segments 8–15 (♂) or 5–10 (♀), a small, mostly sharp tooth until segments 16–19 (♂) or 11–17 (♀) (Fig. 13B, D, E). Epiproct (Fig. 13F, G) conical, flattened dorsoventrally, with two evident apical papillae, both directed ventrocaudally and acute at tip; pre-apical papillae small, lying rather close to tip.

A pair of small, rounded, fully separated, sternal cones between ♂ coxae 4 (Fig. 13H, I). A paramedian pair of evident tubercles in front of gonopod aperture. Legs rather long and slender, faintly incrassate in ♂; midbody legs ca 1.2–1.4 (♂) or 0.9–1.1 (♀) times as long as body height, prefemora without modifications, ♂ tarsal brushes present until legs of segment 8.

**Figure 14.**
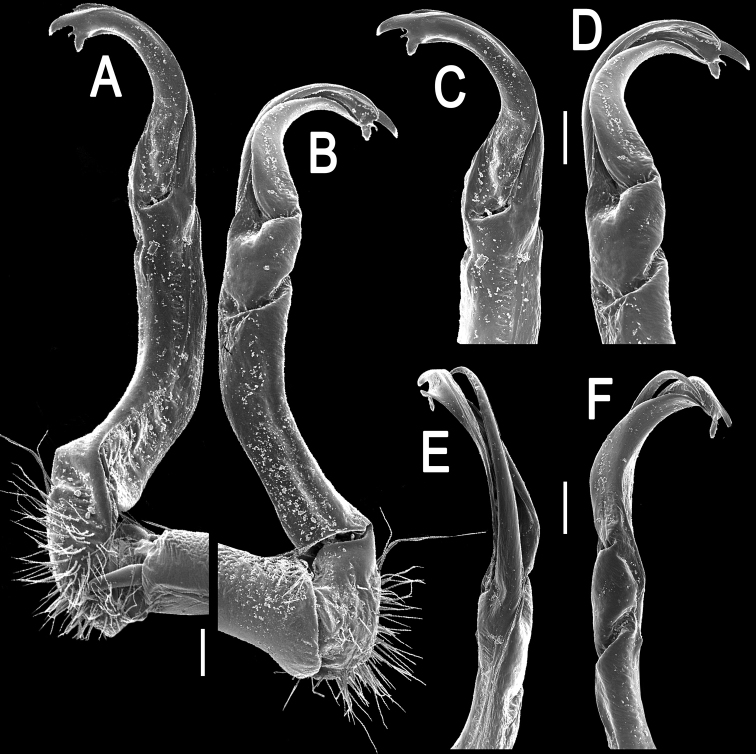
*Orthomorpha sutchariti* sp. n., ♂ holotype, right gonopod. **A, B** mesal and lateral views, respectively **C–F** distal part, mesal, lateral, suboral and subcaudal views, respectively. Scale bar: 0.2 mm.

**Figure 15.**
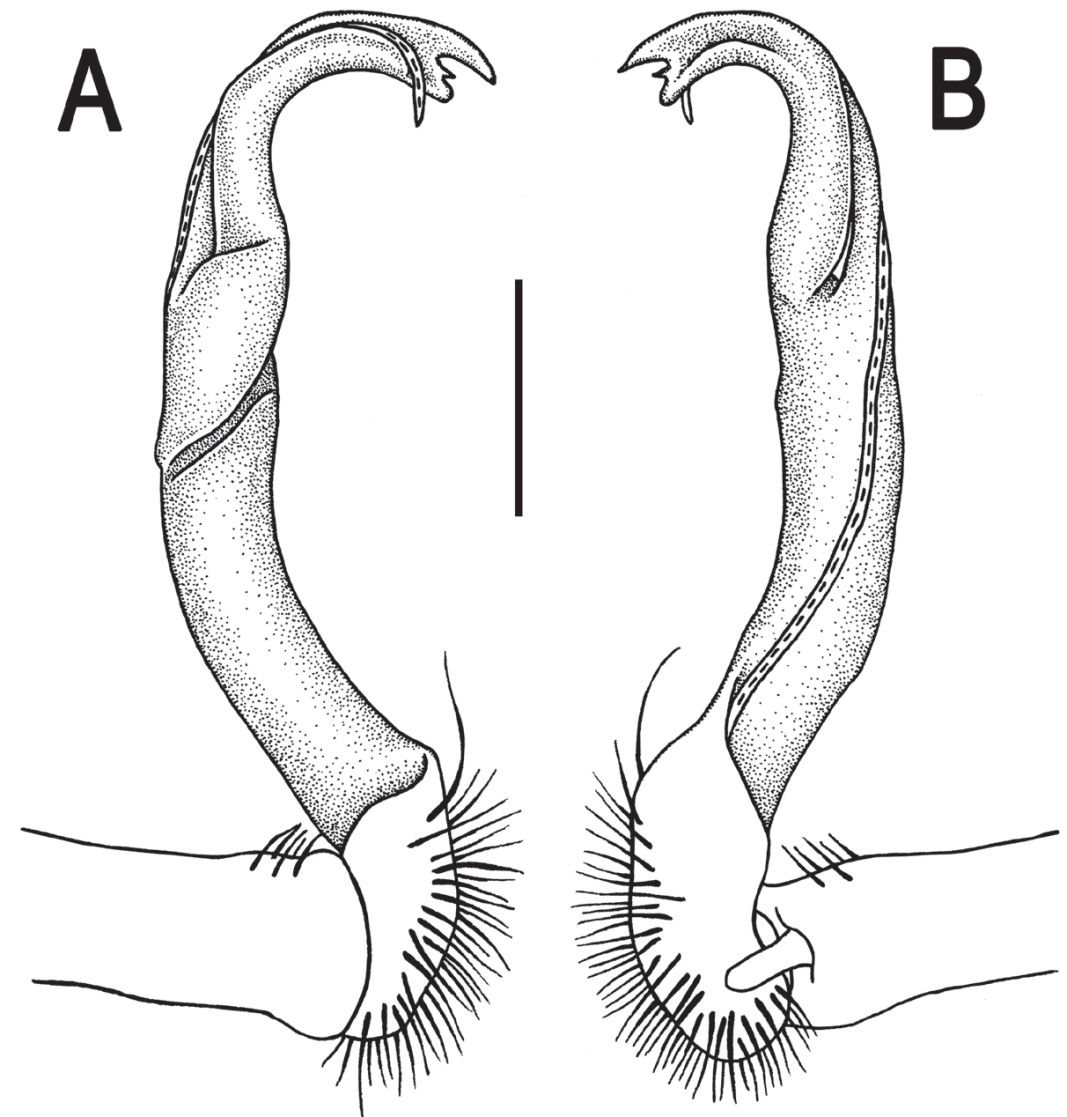
*Orthomorpha sutchariti* sp. n., ♂ holotype. **A**, **B** right gonopod, lateral and mesal views, respectively. Scale bar: 0.5 mm.

Gonopods (Figs 14, 15) with slender and long coxae, the latter with several setae distoventrally. Femorite about 3 times as long as prefemoral portion. Femorite slender, slightly curved, postfemoral portion demarcated by an oblique lateral sulcus; tip of solenophore trifid, with dorsal lobule being subacuminate and longest, middle denticle spiniform, shortest; solenomere long and flagelliform.

##### Remarks.

The colour pattern of *Orthomorpha sutchariti* sp. n. is similar to that observed in several species of the Oriental genus *Antheromorpha* Jeekel, 1968 (our personal observations), but the gonopod structure is typical of *Orthomorpha* spp. Due to the particularly short middle prong at the tip of the solenophore, this new species strongly resembles the numerous congeners within the former *weberi*-group (Likhitrakarn et al. 2011).

## Conclusions

**Figure 16.**
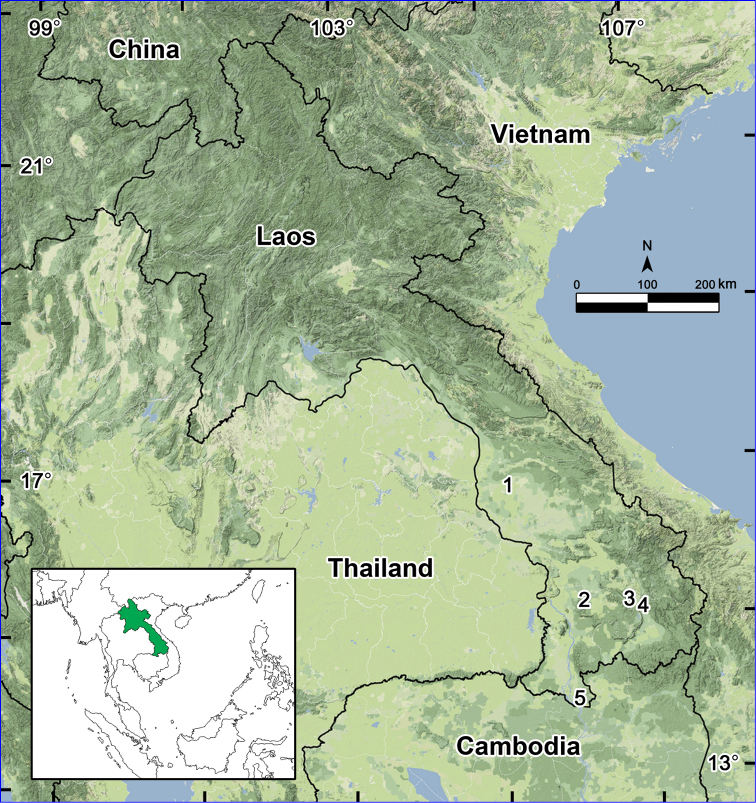
Distributions of the new records of *Orthomorpha* in Laos: **1** Ban Than Bang **2** Tadedu **3** Ban Lak No. 52 **4** Ban Kasom **5** Khone Phapheng Waterfall.

A total of 34 species of millipedes have hitherto been reported from Laos (Likhitrakarn et al. 2014). The present paper adds another three as new to the fauna of Laos, plus another three which are new to science. Since all these new records come from the southern parts of Laos alone (Fig. 16), the generic distribution which shows the northern part of the country as being free from native *Orthomorpha* spp. remains unchanged (Likhitrakarn et al. 2011). There is little doubt that more *Orthomorpha* spp. will be found in countries, like Laos, that are home to this genus.

## Supplementary Material

XML Treatment for Orthomorpha paviei

XML Treatment for Orthomorpha cambodjana

XML Treatment for Orthomorpha communis

XML Treatment for Orthomorpha suberectoides

XML Treatment for Orthomorpha gladiata

XML Treatment for Orthomorpha sutchariti
